# A case of pulmonary vein reconnection after long‐term success of pulmonary vein isolation for atrial fibrillation

**DOI:** 10.1002/joa3.13149

**Published:** 2024-09-19

**Authors:** Kohei Ukita, Yasuyuki Egami, Yasuharu Matsunaga‐Lee, Masamichi Yano, Masami Nishino

**Affiliations:** ^1^ Division of Cardiology Osaka Rosai Hospital Osaka Japan

**Keywords:** atrial fibrillation, catheter ablation, pulmonary vein isolation, pulmonary vein reconnection

## Abstract

We describe a case where right superior pulmonary vein was not reconnected at the beginning of the third radiofrequency catheter ablation (RFCA) for atrial fibrillation but was reconnected at the beginning of the fourth RFCA. This is a case of pulmonary vein reconnection in the chronic phase after successful pulmonary vein isolation.

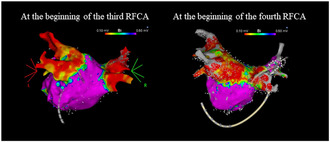

Pulmonary vein (PV) isolation is the cornerstone of catheter ablation (CA) for atrial fibrillation (AF), and durable PV isolation (PVI) is considered to be associated with a lower risk of AF recurrence after CA.^1^ However, the term “durable PVI” has limitations in specifically indicating how many years PVI remains durable. In this report, we present a case of PV reconnection after long‐term success of PVI for AF.

A 71‐year‐old male underwent the fourth radiofrequency CA (RFCA) for recurrent AF. The patient underwent PVI alone during the first RFCA for symptomatic persistent AF at the age of 67 (a 12‐lead electrocardiogram before the RFCA was shown in Figure 1A). The patient did not have significant past medical histories and used rivaroxaban (15 mg/day) and lansoprazole (15 mg/day). The brain natriuretic peptide level was 21.2 pg/mL and the echocardiography showed left atrial enlargement (left atrial diameter: 45 mm).

**FIGURE 1:**
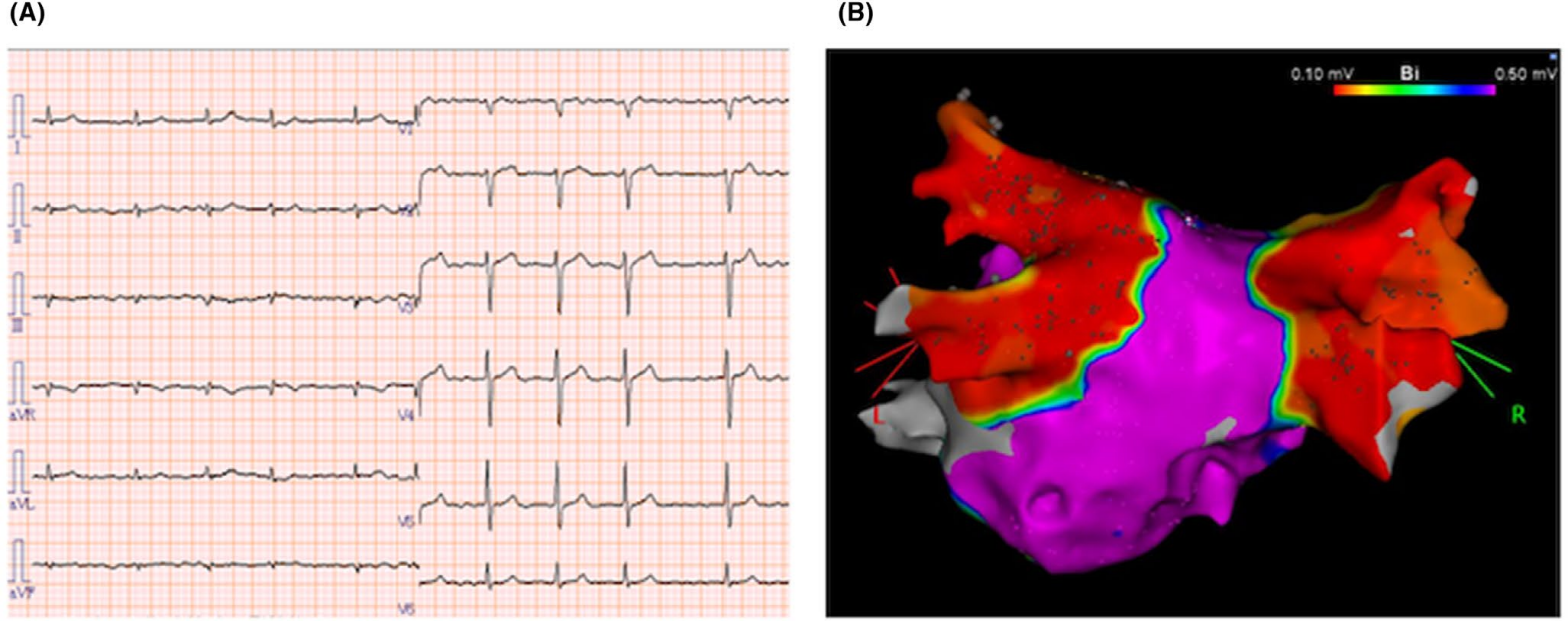
(A) 12‐lead Electrocardiogram before the first RFCA. The heart rate was 83 beats per minute. (B) Voltage map of the LA after the PVI obtained with PENTARAY™ (PA view). 1448 points were acquired. There were no low voltage areas (<0.5 mV) in the LA.

The first RFCA was performed using standard techniques as previously reported.^2^ PVI was achieved with a 3.5 mm ablation catheter with an open‐irrigated tip (THERMOCOOL SMARTTOUCH™ SF, Biosense Webster Inc., Diamond Bar, CA, USA). The ablation was guided by the 3D mapping (CARTO™, Biosense Webster Inc., Diamond Bar, CA, USA) obtained with a 20‐pole mapping catheter (PENTARAY™, Biosense Webster Inc., Diamond Bar, CA, USA). There were no low voltage areas (<0.5 mV) in the left atrium (LA) (Figure 1(B)). Dormant PV conduction was not provoked and AF was not induced by the use of 40 mg of adenosine triphosphate (ATP) under isoproterenol (ISP) infusion (2 μg/min). At the end of the study, the bidirectional conduction block between the LA and PVs was confirmed.

Eight months later, the second RFCA was performed due to the recurrence of AF. At the beginning of the procedure, each PV was assessed with PENTARAY™. The right superior PV (RSPV) was reconnected and re‐isolated by the radiofrequency application to the posterior carina of the RSPV (Figure 2A). In addition, superior vena cava (SVC) isolation was performed. Both RSPV and SVC were not reconnected and non‐PV foci were not detected by the use of 40 mg of ATP under ISP infusion (4 μg/min).

**FIGURE 2:**
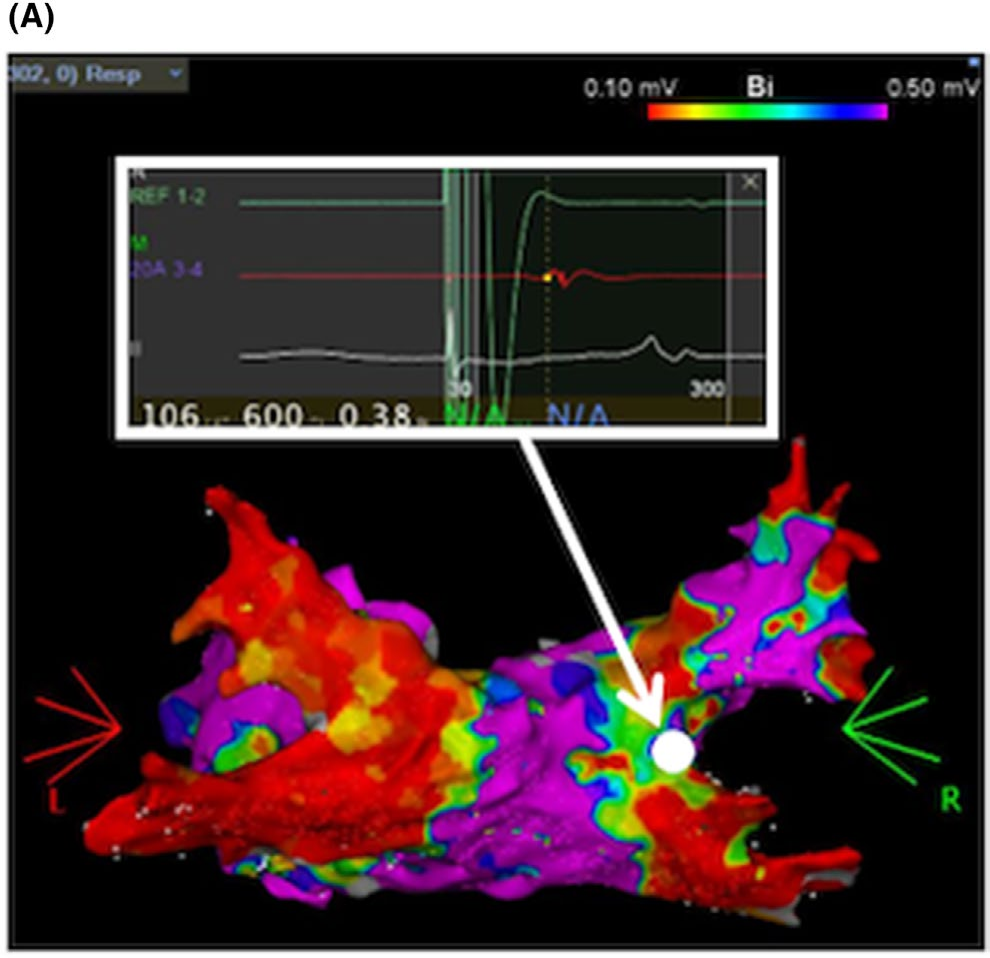
(A) Voltage map of the LA at the beginning of the second RFCA obtained with PENTARAY™ (PA‐cranial view). 2302 points were acquired. The RSPV was reconnected and re‐isolated by the radiofrequency application to the posterior carina of the RSPV (white circle).

Six months later, the third RFCA was performed due to the recurrence of AF. Each PV was assessed with PENTARAY™ as well as in the second RFCA, and there were no PV reconnections (Figure 3A). Dissociated potentials were reproducibly shown in the RSPV (Figure 3B). Furthermore, the absence of conduction into the LA from the RSPV was confirmed by the pacing from the RSPV. Furthermore, there were no SVC reconnections. Subsequently, LA posterior wall (LAPW) isolation was performed. The voltage map after the LAPW isolation was shown in Figure 3C. No reconnections of PV, SVC, or LAPW were observed by the use of 40 mg of ATP under ISP infusion (4 μg/min).

**FIGURE 3:**
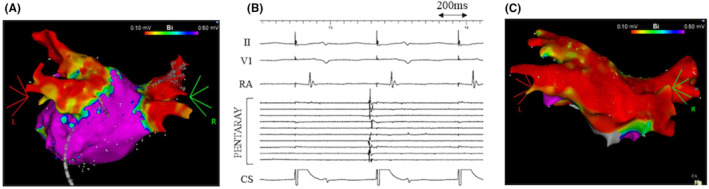
(A) Voltage map of the LA at the beginning of the third RFCA obtained with PENTARAY™ (PA view). 2426 points were acquired. There were no PV reconnections. (B) Intracardiac electrogram when PENTARAY™ was located in the RSPV. Dissociated potentials were reproducibly shown in PENTARAY™. (C) Voltage map after LAPW isolation obtained with PENTARAY™ (PA view). CS, coronary sinus; RA, right atrium.

Nevertheless, recurrent symptomatic AF was detected 6 months after the third RFCA. The recurrence pattern was paroxysmal AF initially, but AF progressed from paroxysmal to persistent and the symptoms worsened, resulting in performing the fourth RFCA. At the beginning of the fourth RFCA (34 months after the third RFCA), each PV was assessed with a multipolar mapping catheter (OCTARAY™, Biosense Webster Inc., Diamond Bar, CA, USA). The RSPV was considered to be reconnected (Figure 4A) and re‐isolated by the radiofrequency application to the posterior carina of the RSPV, which was almost the same site as in the second RFCA (Figure 4B). In addition, the LAPW was also reconnected and re‐isolated by the radiofrequency application to the middle of the LA roof line. RSPV firing was reproducibly observed by the use of 40 mg of ATP under ISP infusion (4 μg/min), which indicated arrhythmogenesis in the RSPV (Figure 4C).

**FIGURE 4:**
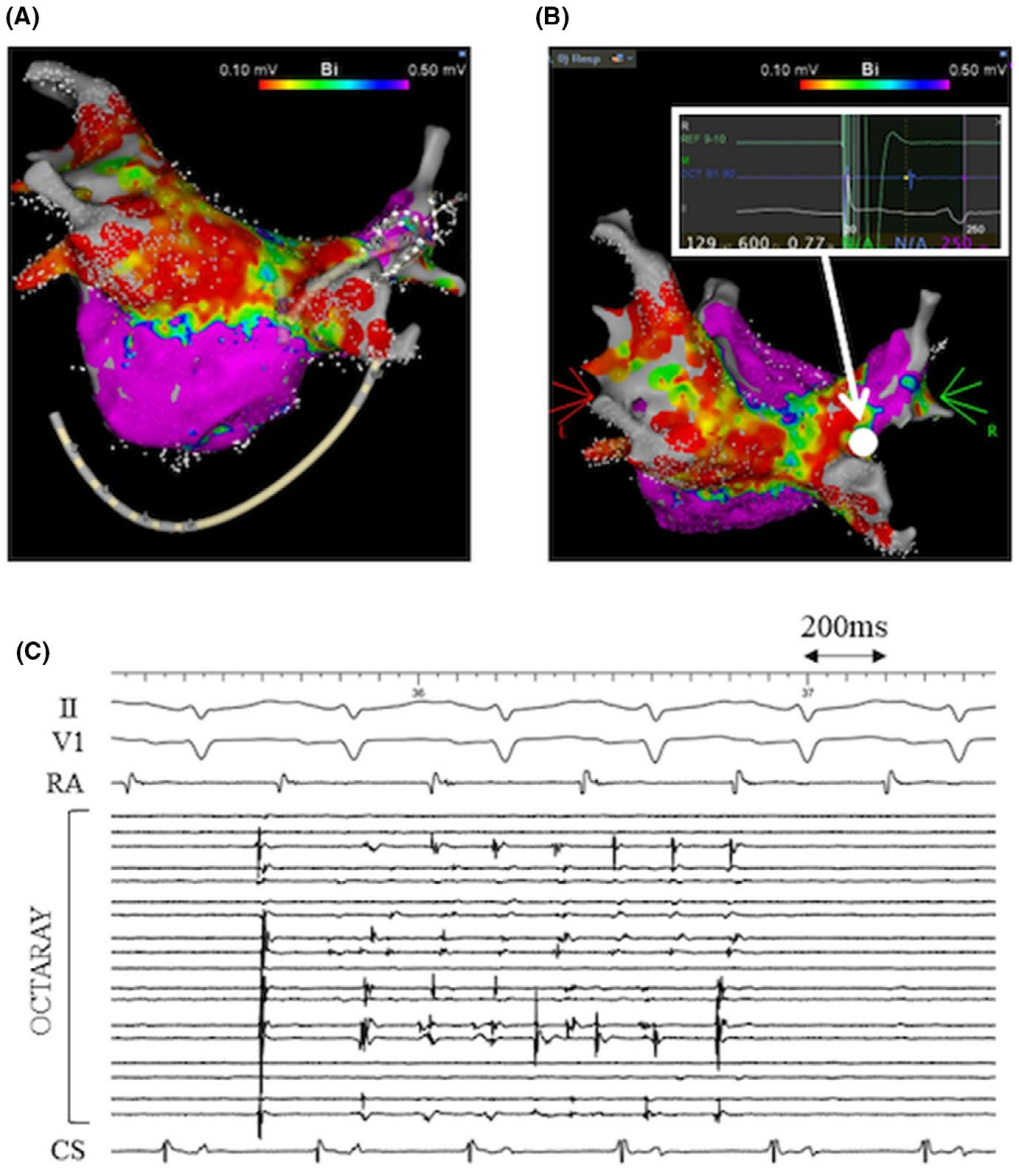
(A) Voltage map of the LA at the beginning of the fourth RFCA obtained with OCTARAY™ (PA view). 4519 points were acquired. The RSPV was reconnected. (B) Voltage map of the LA at the beginning of the fourth RFCA obtained with OCTARAY™ (PA‐cranial view). The RSPV was re‐isolated by the radiofrequency application to the posterior carina of the RSPV (white circle), which was almost the same site as in the second RFCA. (C) Intracardiac electrograms after re‐isolation of the RSPV. OCTARAY™ was located in the RSPV. RSPV firing was reproducibly observed by the use of 40 mg of ATP under ISP infusion (4 μg/min). CS, coronary sinus; RA, right atrium.

The patient has been doing well without symptoms or recurrences of atrial tachyarrhythmia for more than 1 year since the fourth RFCA.

In this case, RSPV reconnection was not observed at the beginning of the third RFCA but was detected at the beginning of the fourth RFCA. In other words, durable PVI was achieved for at least 6 months (from the second to the third RFCA) but the RSPV reconnected at some point during the subsequent 34 months (from the third to the fourth RFCA).

A case was previously reported showing dormant PV conduction revealed by ATP after confirming no PV potentials at the initial CA for AF.^3^ In our case, however, dormant PV conduction was not observed by ATP during the third RFCA. Regarding the pathological mechanisms of PV reconnection, it has been reported that tissue within PVI scar exhibited nuclear pyknosis and myocytolysis even months to years after ablation, indicating that tissue can remain viable and potentially recover in the late phase after ablation.^4^ Tissue remodeling may also contribute to delayed PV reconnection. This case suggests that a repeat ablation procedure might be one option considering PV reconnection even after multiple procedures, especially in the case where the presence of non‐PV foci seems unlikely.

It may be hard to identify retrospectively the cause of recurrence of AF before the third RFCA. Judging from the fact that the first recurrence of AF was detected on the second day after the second RFCA and AF sustained until the beginning of the third RFCA, the cause of AF recurrence after the second RFCA might be related to the inflammatory response or imbalance of the autonomic nervous system and the recurrence might have affected the post‐ablation remodeling and resulted in recurrence as persistent AF before the third RFCA.^5^

There are several limitations in this report. First, there was a difference in the mapping catheter between the third and the fourth RFCA (PENTARAY™ and OCTARAY™, respectively). Second, circular mapping catheters, which might have been more appropriate for confirming PVI, were not used in the third RFCA. Third, we cannot completely rule out the possibility that the first manipulation of the PENTARAY™ into the RSPV blocked the barely existing PV‐LA conduction at the beginning of the third RFCA and the RSPV was not reconnected during the procedure.

## CONFLICT OF INTEREST STATEMENT

The authors declare that there is no conflict of interest.

## ETHICS STATEMENT

This study was approved by the Osaka Rosai Hospital Ethics Committee.

## CONSENT

The patient provided written informed consent for the ablation procedures and agreed to the publication of his case details and images in this report.

## Data Availability

The data that support the findings of this study are available from the corresponding author upon reasonable request.
